# Supplementary figures and images for: Genetic risk scores and dementia risk across different ethnic groups in UK Biobank
Source: PLoS One. 2022 Dec 7;17(12):e0277378. doi: 10.1371/journal.pone.0277378 (PMC9728885; doi:10.1371/journal.pone.0277378)

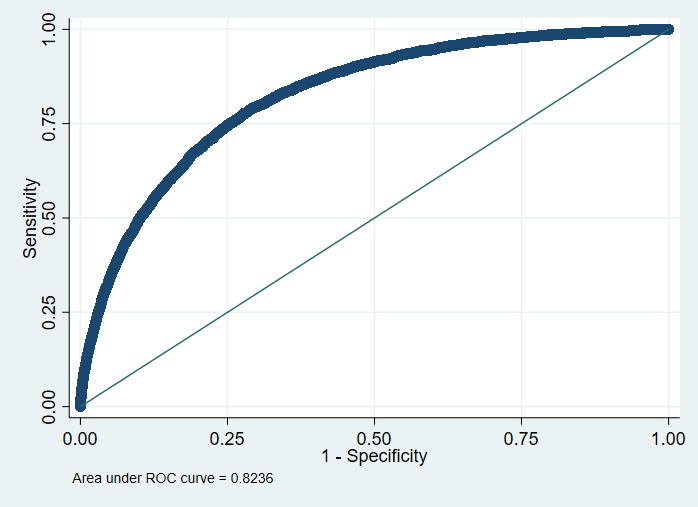

Supplement: S1 Fig — (TIF) [file pone.0277378.s001.tif]

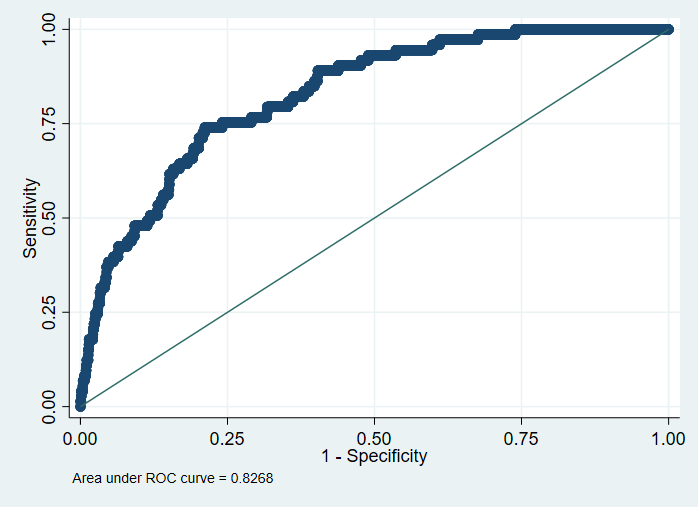

Supplement: S2 Fig — (TIF) [file pone.0277378.s002.tif]

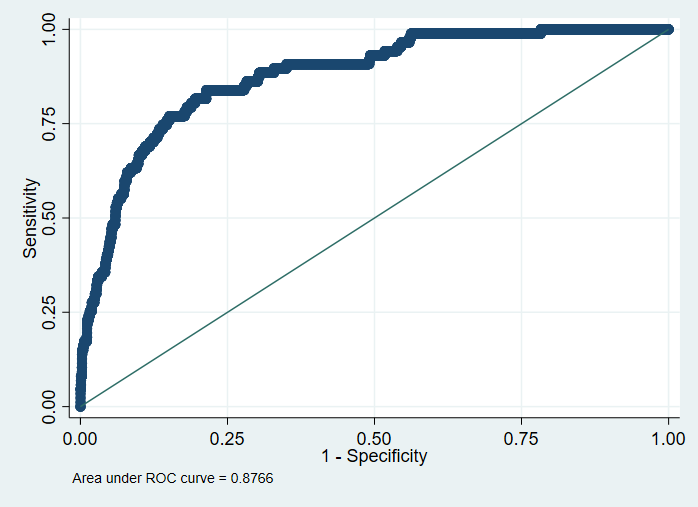

Supplement: S3 Fig — (TIF) [file pone.0277378.s003.tif]
