# Supplementary material for: Genetic risk scores and dementia risk across different ethnic groups in UK Biobank
Source: PLoS One. 2022 Dec 7;17(12):e0277378. doi: 10.1371/journal.pone.0277378 (PMC9728885; doi:10.1371/journal.pone.0277378)
Supplement: S1 File — (DOCX) [file pone.0277378.s004.docx]

# Genetic risk scores and dementia risk across different ethnic groups in UK Biobank: Supplementary material

# Table S1: APOE

We determined APOE genotype based on the following combinations of rs429358 and rs7412 SNPs.

| APOE genotype | rs429358 | rs7412 | ε2 alleles | ε4 alleles |
| --- | --- | --- | --- | --- |
| ε1/ε1 | C;C | T;T | 0 | 0 |
| ε1/ε2 | C;T | T;T | 1 | 0 |
| ε1/ε3 or ε2/ε4 | C;T | C;T | 1* | 1* |
| ε1/ε4 | C;C | C;T | 0 | 1 |
| ε2/ε2 | T;T | T;T | 2 | 0 |
| ε2/ε3 | T;T | C;T | 1 | 0 |
| ε3/ε3 | T;T | C;C | 0 | 0 |
| ε3/ε4 | C;T | C;C | 0 | 1 |
| ε4/ε4 | C;C | C;C | 0 | 2 |

* ε1 is a rare allele so we determined this genotype to be more likely ε2/ε4 than ε1/ε3

# Genetic risk score calculation

The formula we used for calculating genetic risk score is shown below:

GRS $=\frac{\sum_{j=1}^{m} X_{j}\beta_{j}}{m}$

Where X is the genotype dosage at a particular location (0, 1 or 2), j denotes the SNP identity, β is the weight at that SNP (log odds ratio for the SNP with regards to dementia) and m is the number of SNPs used in the calculation.

# Table S2: SNPs and log odds ratios used in GRS calculation

| Chromosome | Base position^¥^ | SNP | Effect allele | Nearest gene* | Log OR^₸^ |
| --- | --- | --- | --- | --- | --- |
| 1 | 207692049 | rs6656401 | A | CR1 | 0.165514 |
| 2 | 127892810 | rs6733839 | T | BIN1 | 0.198851 |
| 2 | 234068476 | rs35349669 | T | INPP5D | 0.076961 |
| 5 | 88223420 | rs190982 | G | MEF2C | -0.07257 |
| 6 | 32578530 | rs9271192 | C | HLA-DRB1 | 0.10436 |
| 6 | 47487762 | rs10948363 | G | CD2AP | 0.09531 |
| 7 | 37841534 | rs2718058 | G | NME8 | -0.07257 |
| 7 | 100004446 | rs1476679 | C | ZCWPW1 | -0.09431 |
| 7 | 143110762 | rs11771145 | A | EPHA1 | -0.10536 |
| 8 | 27195121 | rs28834970 | C | PTK2B | 0.09531 |
| 8 | 27467686 | rs9331896 | C | CLU | -0.15082 |
| 11 | 47557871 | rs10838725 | C | CELF1 | 0.076961 |
| 11 | 59923508 | rs983392 | G | MS4A6A | -0.10536 |
| 11 | 85867875 | rs10792832 | A | PICALM | -0.13926 |
| 11 | 121435587 | rs11218343 | C | SORL1 | -0.26136 |
| 14 | 53400629 | rs17125944 | C | FERMT2 | 0.131028 |
| 14 | 92926952 | rs10498633 | T | SLC24A4  RIN3 | -0.09431 |
| 19 | 1063443 | rs4147929 | A | ABCA7 | 0.139762 |
| 19 | 45411941 | rs429358 | T | APOE | -0.47 (ε2)  1.03 (ε4) |
| 19 | 45412079 | rs7412 | C | APOE |  |
| 20 | 55018260 | rs7274581 | C | CASS4 | -0.12783 |

^¥^Build 37, assembly hg19 *Genes located +/- 100 kb of the top SNP. ^₸^All log odds ratios and details about SNPs are from Lambert et al^1^.

# Table S3: Minor allele frequency (MAF) and Info score for SNPs included in analysis

|  |  | **European ancestry** | | **Non-European ancestry** | |
| --- | --- | --- | --- | --- | --- |
| **rsid** | **Nearest gene** | **MAF** | **Info score** | **MAF** | **Info score** |
| rs6656401 | CR1 | 0.172162 | 0.999121 | 0.102191 | 0.997662 |
| rs6733839 | BIN1 | 0.390541 | 0.96347 | 0.383342 | 0.907574 |
| rs35349669 | INPP5D | 0.482506 | 0.996789 | 0.266989 | 0.988194 |
| rs190982 | MEF2C | 0.391445 | 0.967398 | 0.302336 | 0.940597 |
| rs9271192 | HLA-DRB1 | 0.273599 | 0.999773 | 0.242209 | 0.999802 |
| rs10948363 | CD2AP | 0.271169 | 0.999231 | 0.210825 | 0.997452 |
| rs2718058 | NME8 | 0.362645 | 0.993747 | 0.338552 | 0.983832 |
| rs1476679 | ZCWPW1 | 0.301832 | 0.997381 | 0.205056 | 0.98671 |
| rs11771145 | EPHA1 | 0.35129 | 1 | 0.390487 | 1 |
| rs28834970 | PTK2B | 0.365349 | 0.996214 | 0.361392 | 0.987005 |
| rs9331896 | CLU | 0.411791 | 0.989543 | 0.412613 | 0.969667 |
| rs10838725 | CELF1 | 0.299947 | 0.999387 | 0.292839 | 0.998487 |
| rs983392 | MS4A6A | 0.401169 | 0.994394 | 0.318402 | 0.974139 |
| rs10792832 | PICALM | 0.365831 | 0.999258 | 0.315952 | 0.997387 |
| rs11218343 | SORL1 | 0.040436 | 0.992253 | 0.080286 | 0.968019 |
| rs17125944 | FERMT2 | 0.09192 | 0.991601 | 0.089905 | 0.967295 |
| rs10498633 | SLC24A4  RIN3 | 0.226713 | 1 | 0.178001 | 1 |
| rs4147929 | ABCA7 | 0.173605 | 0.997541 | 0.149672 | 0.993595 |
| rs429358 | APOE | 0.15407 | 1 | 0.140253 | 1 |
| rs7412 | APOE | 0.07999 | 1 | 0.073903 | 1 |
| rs7274581 | CASS4 | 0.08597 | 0.986035 | 0.106356 | 0.968783 |

#

# Table S4: Regression analyses for secondary outcomes with z-GRS excluding APOE as exposure

| **Dependent variable** | **Main effect** | | | | **Interaction effect, South Asian participants, Coeff/OR (95%CI, p-value)** | **Interaction effect, Black participants, Coeff/OR (95%CI, p-value)** |
| --- | --- | --- | --- | --- | --- | --- |
|  | **Coeff/OR*** | **LCI** | **UCI** | **p-value** |  |  |
| Family history of dementia | 1.10 | 1.09 | 1.11 | <0.0001 | 0.95(0.85-1.07, 0.439) | 0.97 (0.86-1.10, 0.689) |
| Reaction time | -0.05 | -0.40 | 0.31 | 0.80 | 2.54(-0.33 to 5.42, 0.083) | -0.23 (-3.51 to 3.05, 0.892) |
| Hippocampal volume | -10.69 | -18.06 | -3.31 | 0.01 | 20.55(-54.91 to 96.01, 0.594) | 67.75 (-36.62 to 172.11, 0.203 |
| Amygdala volume | -3.07 | -6.84 | 0.72 | 0.11 | 36.79 (-1.89 to 75.46, 0.0.6) | 16.00 (-37.49 to 69.50, 0.558) |

*** Coefficient for linear regression and Odds ratio for logistic regression after age, sex, genetic ancestry and genetic principal component adjustment (and brain volume for hippocampal and amygdala volume regressions), LCI=Lower confidence interval, UCI=Upper confidence interval**

# Table S5: Baseline characteristics of participants by genetic ancestry grouping

| **Characteristic** |  | **White British (N=368,277)** | **South Asian (N=7,869)** | **Black**  **(N=8,413)** |
| --- | --- | --- | --- | --- |
| Age (mean, SD) |  | 56.8 (8.0) | 53.6 (8.5) | 51.8 (8.1) |
| N(%) female |  | 198,200 (53.8) | 3,641 (46.3) | 4,817 (57.3) |
| Family history of dementia N(%) | None | 321,178 (87.2) | 7,503 (95.4) | 7,934 (94.3) |
|  | One parent | 45,176 (12.3) | 348 (4.4) | 464 (5.5) |
|  | Both parents | 1,923 (0.5) | 18 (0.2) | 15 (0.2) |
| ε2 allele N(%) | 0 | 320,807 (87.1) | 7,296 (92.7) | 7,125 (84.7) |
|  | 1 | 45,160 (12.3) | 559 (7.1) | 1,189 (14.1) |
|  | 2 | 2,314 (0.6) | 14 (0.2) | 101 (1.2) |
| ε4 allele N(%) | 0 | 309,826 (76.2) | 6,937 (83.5) | 6,128 (68.2) |
|  | 1 | 89,054 (21.9) | 1,298 (15.6) | 2,434 (27.1) |
|  | 2 | 7,936 (2.0) | 74 (0.9) | 426 (4.7) |
| Reaction time in seconds - mean(SD) |  | 557 (114) | 616 (155) | 632 (179) |
| Dementia cases N(%) |  | 4,768 (1.3%) | 91 (1.2%) | 105 (1.3%) |
| Mean z-standardised PRS score including APOE (SD) |  | 0.00(1.00) | -0.14(0.80) | 0.24(1.19) |
| Mean z-standardised PRS score excluding APOE (SD) |  | 0.01(1.00) | 0.14(0.97) | -0.49(0.82) |
|  |  | **N= 32,849** | **N= 375** | **N= 286** |
| Hippocampal volume - left plus right (Mean(SD) in mm^3^) |  | 8066.0 (804.8) | 7918.8 (846.6) | 7956.3 (754.3)) |
| Amygdala volume - left plus right (Mean(SD) in mm^3^) |  | 3289.3 (425.9) | 3264.0 (457.0) | 3254.8 (412.9) |
| Brain volume, normalised for head size (Mean(SD) in mm^3*^10^6^) |  | 1.5(0.07) | 1.5(0.08) | 1.5(0.07) |

# Table S6: Dementia risk associated with genotype by genetic ancestry grouping

|  |  | **Main effect** | | | | **Interaction effect, South Asian participants, Coeff/OR (95%CI, p-value)** |  |
| --- | --- | --- | --- | --- | --- | --- | --- |
| **Exposure** |  | **OR*** | **LCI** | **UCI** | **p value** |  | **Interaction effect, Black participants, Coeff/OR (95%CI, p-value)** |
| ε2 alleles | 0 | Reference | | | | OR 0.99 (0.36-2.70, 0.978) | OR 1.01 (0.53-1.93, 0.974) |
|  | 1 | 0.54 | 0.48 | 0.60 | <0.0001 |  |  |
|  | 2 | 0.56 | 0.35 | 0.89 | 0.014 |  |  |
| ε4 alleles | 0 | Reference | | | | OR 1.02 (0.71-1.45, 0.932) | OR 0.88 (0.66-1.18, 0.401) |
|  | 1 | 2.57 | 2.42 | 2.73 | <0.0001 |  |  |
|  | 2 | 8.44 | 7.66 | 9.31 | <0.0001 |  |  |
| z-GRS with APOE - effect per SD increase |  | 1.72 | 1.68 | 1.76 | <0.0001 | OR 1.04 (0.85-1.26, 0.722) | OR 0.92 (0.79 – 1.08, 0.333) |
| z-GRS without APOE – effect per SD increase |  | 1.21 | 1.18 | 1.24 | <0.0001 | OR 0.86 (0.69-1.07, 0.163) | OR 0.93 (0.74-1.19, 0.577) |

***Odds ratio of dementia, adjusted for age, sex, ancestry, genetic principal components. SD=Standard deviation LCI=Lower confidence interval UCI=Upper Confidence interval. Interaction tested between main exposure and genetic ancestry for association with dementia**

# Table S7: Regression analyses for secondary outcomes by genetic ancestry grouping

| **Dependent variable** | **Main effect** | | | | **Interaction effect Coefficient/OR (95%CI, p-value)** |
| --- | --- | --- | --- | --- | --- |
|  | **Coeff/OR*** | **LCI** | **UCI** | **p-value** |  |
| Family history of dementia | 1.32 | 1.30 | 1.33 | <0.0001 | 0.98 (0.96-1.00, 0.056) |
| Reaction time | 0.27 | -0.08 | 0.62 | 0.13 | 0.58 (-0.12 to 1.27, 0.104) |
| Hippocampal volume | -7.14 | -14.74 | 0.46 | 0.07 | 5.23 (-13.55 to 24.02, 0.585) |
| Amygdala volume | -2.64 | -6.53 | 1.25 | 0.18 | -2.89 (-12.51 to 6.73, 0.555) |

***Coefficient for linear regression and OR for logistic regression after age, sex, ancestry and genetic principal component adjustment (and brain volume for hippocampal and amygdala volume regressions), LCI=Lower confidence interval, UCI=Upper confidence interval**

# References

1. Lambert JC, Ibrahim-Verbaas CA, Harold D, et al. Meta-analysis of 74,046 individuals identifies 11 new susceptibility loci for Alzheimer's disease. *Nature genetics* 2013; **45**(12): 1452-8.
