# Supplementary material for: Genetic risk scores and dementia risk across different ethnic groups in UK Biobank
Source: PLoS One. 2022 Dec 7;17(12):e0277378. doi: 10.1371/journal.pone.0277378 (PMC9728885; doi:10.1371/journal.pone.0277378)
Supplement: S2 File — (PDF) [file pone.0277378.s005.pdf]

Non-European dataset in UK Biobank (N= 38598)

Files included in this folder:

- First 32 principal components (PCs) calculated for the non-European dataset (computed with GENESIS package)
- Eigenvalues of the provided PCs (computed with GENESIS package)
- List of the up to 3rd-degree relatives in the non-European dataset and the corresponding kinship values (computed with GENESIS package)
- Summary statistic files (MAF, INFO information, computed with SNPTEST)

The participant IDs in all the provided files come from project 9922.

We have also identified samples belonging to the following ancestry groups based on genetic data: East Asian N=2464, South Asian N=8964, Black N=9233, Admixed with predominantly European origin (N=11251). We can share this information upon request.

1.1. Non-European dataset definition

38,598 participants were considered in the non-European UK Biobank dataset after exclusion of gender mismatches, missingness/heterozygosity outliers, participants with excessive genetic relatedness, no QC metrics, individuals that have withdrawn their consent (based on sample-QC information provided by UK Biobank team) and European participants (samples with UK Biobank provided PC1<0 and PC2 > -10, green lines in Figure *1*). The initial selection of European and non-European samples was done by Dr. Alina Farmaki, as described in Figure *1*.

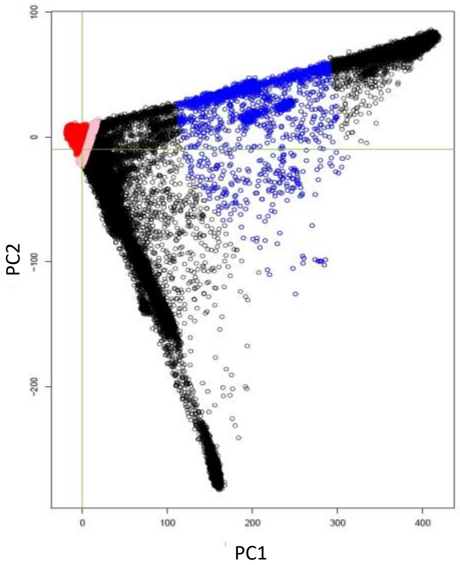

Figure 1. Definition of European and non-European samples in the UK Biobank dataset. Samples with PC1< 0 and PC2> -10 (as provided by UK Biobank team) were defined as European. The corresponding values for White British were PC1< -6.05 and PC2 > -2.02). The red colour depicts the EUR (~450,000) as reflected by the k-means 7 and the pink colour depicts the EUR (~456,000) as reflected by the k-means 6.

1.2. Population Structure and Relatedness Inference using the GENESIS package (Matthew P.Conomos, 2019-02-20, R version 3.5)

GENESIS provides statistical methodology for analysing genetic data from samples with population structure and/or familial relatedness.

This analysis was based on a subset of 171,258 SNPs of the non-European dataset (implying the same thresholds with UK Biobank PCA analysis, suppl. file of Bycroft et al., including missing rate > 0.015, MAF <0.01 , markers in regions of long-range LD as provided by UK Biobank team and pruning to a set of independent markers such that pairwise r2 < 0.1, using windows of 1000 markers and a step-size of 80 markers).

Two rounds of principal components and relatedness calculation were performed in this project:

- Principal component analysis was performed with PC-AiR algorithm that accounts for known or cryptic relatedness, to get PCs that capture population and not family structure (Figure 2) (Conomos, Miller, & Thornton, 2015). The first 32 PCs and corresponding eigenvalues are provided.
- Relatedness was estimated again using the more precise PCs using the PC-Relate method (Conomos, Reiner, Weir, & Thornton, 2016). The list of up to 3rd-degree relatives based on the relatedness estimation adjusted for PCs and the corresponding kinship values is provided. There was a significant reclassification of individuals based on their relatedness compared to UK Biobank provided metrics (Table 1).

Table 1. Number of related pairs based on the UK Biobank provided (KING) and the PC-Relate calculated kinships.

	**UK Biobank provided Kinship**				
**PC-Relate Kinship**	duplicates/MZ twins (N=11)	1st degree (N=1519)	2nd degree (N=695)	3rd degree (N=2374)	Unrelated (N=564)
duplicates/MZ twins (N=14)	11	3	-	-	
1st degree (N=1320)	-	1314	5	1	-
2nd degree (N=643)	-	1	482	96	64
3rd degree (N=1683)	-	-	9	1176	498
Unrelated (N=1503)	-	201	199	1101	2

an estimated kinship coefficient range >0.354, [0.177, 0.354], [0.0884, 0.177] and [0.0442, 0.0884] corresponds to duplicate/MZ twin, 1st-degree, 2nd-degree, and 3rd-degree relationships respectively

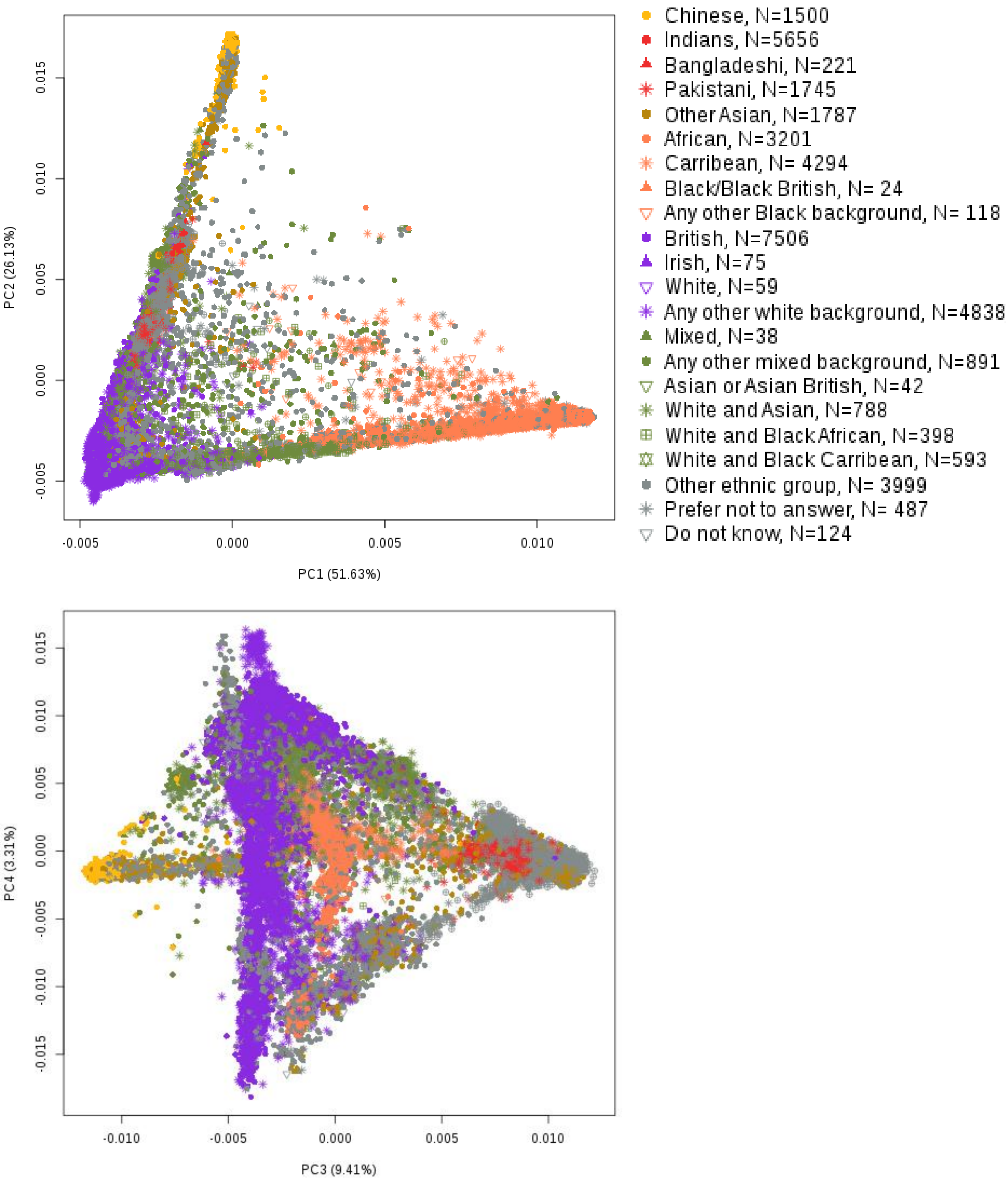

Figure 2. First 4 principal components in PCA on non-European UK Biobank dataset (PC-AiR), coloured according to their self-report ethnic background.

1.3. Summary statistics (computed with SNPTEST)

- Non-Europeans (Minor Allele Frequency, INFO score): two analyses were performed; one with the whole dataset and one after excluding second degree relatives (based on kinship information provided by UK Biobank)
- Europeans (Minor Allele Frequency, INFO score, Hardy-Weinberg Equilibrium): two analyses were performed for the related and the unrelated subsets as above

References

Conomos, M. P., Miller, M. B., & Thornton, T. A. (2015). Robust inference of population structure for ancestry prediction and correction of stratification in the presence of relatedness. *Genet Epidemiol, 39*(4), 276-293. doi:10.1002/gepi.21896

Conomos, M. P., Reiner, A. P., Weir, B. S., & Thornton, T. A. (2016). Model-free Estimation of Recent Genetic Relatedness. Am J Hum Genet, 98(1), 127-148. doi:10.1016/j.ajhg.2015.11.022
